# Upcycling birch bark suberin into versatile and recyclable thermosets

**DOI:** 10.1039/d5gc06834g

**Published:** 2026-03-17

**Authors:** Fengyang Wang, Ruslan Gryaznov, Pavel Vostrejs, Matilda Andersson, Jānis Rižikovs, Ievgen Pylypchuk, Alberto J. Huertas-Alonso, Mika H. Sipponen

**Affiliations:** a Department of Chemistry, Stockholm University SE-10691 Stockholm Sweden albertojose.huertasalonso@su.se mika.sipponen@su.se; b Department of Chemistry, Wallenberg Wood Science Center, Stockholm University SE-10691 Stockholm Sweden; c Latvian State Institute of Wood Chemistry, Biorefinery Laboratory Latvia

## Abstract

There is an urgent need for recyclable thermosets from renewable feedstocks that can be precisely tailored for diverse applications. Here we show that birch outer bark, an abundant forestry by-product, can be converted into fully biobased thermosets with tunable mechanical properties and closed-loop recyclability. Catalyst-free crosslinking of suberin-derived precursors yields materials spanning from elastic adhesives to stiff anticorrosion coatings and carbon fiber composites. These thermosets undergo facile chemical recycling *via* alkaline hydrolysis and can be thermally repolymerized, exhibiting progressively enhanced strength across multiple cycles. After five recycling rounds, tensile strength increases from 0.5 MPa at 200% strain to 26 MPa at 98% strain, with material recovery yields above 90% at each cycle. This unusual feature was ascribed to ester/ether exchange and enables the preparation of recyclable suberinic acids (SAs)/carbon fiber reinforced polymer (CFRP), where the fiber and polymer properties are not compromised after the recycling process. Overall, the fully biobased thermosets developed in this study can match or even exceed the performance of fossil-based counterparts while providing true circular-economy benefits.

## Abstract 2

Suberinic acids from birch bark are converted into robust, fully recyclable thermosets with control over crosslink density *via* reversible ester chemistry, enabling a scalable route to circular high-performance materials from forestry residues.

## Graphical abstract

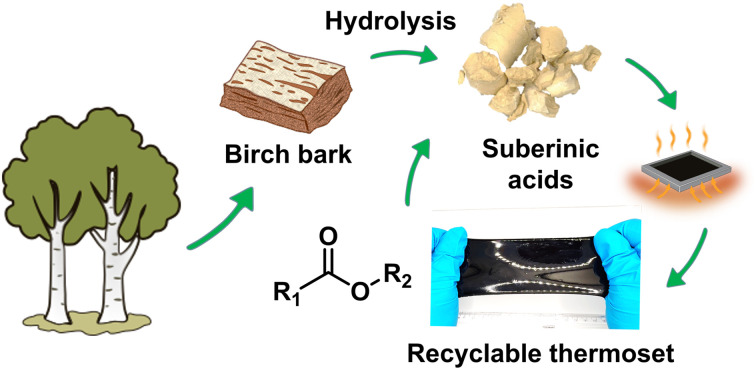

**Green foundation**

1. This work converts birch bark-derived suberinic acids into fully bio-based thermosets that retain reprocessability through reversible ester linkages, demonstrating a sustainable alternative to petroleum-based crosslinked polymers and upgrading a forestry side-stream into high-value materials.

2. The thermoset is developed exclusively from birch outer bark, which is polymerized through step-growth condensation in the melt without solvent or toxic crosslinkers. The resulting thermoset can be recycled and reprocessed multiple times while maintaining high mechanical performance. Its curing chemistry avoids metal catalyst, reducing environmental and safety impacts.

3. Future developments may focus on lowering curing temperatures, optimizing bark fractionation to reduce energy use, and tuning crosslink density and properties for targeted applications at scale, while assessing environmental and economic viability.

## 1. Introduction

More than 60 million tons of petroleum-derived thermosets are produced annually, with most discarded through landfilling or combustion at end of life.^1^ Biobased thermosets are covalently crosslinked polymers from renewable feedstocks that promise sustainable alternatives to fossil-derived counterparts.^2^ In practice, progress has been limited because natural precursors often require complex fractionation and chemical activation, and petroleum-derived crosslinkers are still incorporated to achieve desirable processing and performance, particularly in adhesives, composites, and coatings.^3–5^ Fully biobased thermosets that combine tunable properties with recyclability and circularity remain rare.

Birch bark, largely treated as a low-value by-product in forestry and typically combusted for energy, represents an untapped feedstock. Its outer layer contains suberin, a natural crosslinked polyester that confers water resistance and mechanical durability,^6^ properties that have historically enabled its use in long-lasting Scandinavian sod roofs.^7^ Building on this inspiration, we explored alkaline hydrolysis of birch outer bark as a route to recyclable thermosets. The resulting suberinic acids (SAs) comprise a complex mixture of long-chain aliphatic acids (hydroxy acids, polyunsaturated fatty acids, diacids) along with betulin and ferulic acid.^8,9^ While prior studies have demonstrated crosslinking of SAs,^10–12^ the underlying reaction pathways and process–structure–property relationships remain unresolved.

Here we show, through a combination of chemical, spectroscopic, and mechanical analyses, how curing time governs the evolution of crosslinking density, enabling continuous tuning of material properties from highly flexible elastomers to stiff dimensionally stable thermosets. This structure–property control over network formation provides a straightforward but powerful means to engineer performance on demand without additional chemical modification. Remarkably, these materials retain and even show largely enhanced mechanical strength across five cycles of chemical recycling, underscoring the robustness of the underlying reversible ester network.

By elucidating the molecular and crosslinking mechanisms that dictate the performance of SAs based thermosets, our work establishes scalable routes to fully biobased and closed-loop recyclable polymers, elevating birch bark from an underutilized forestry residue to a high-value renewable feedstock. More broadly, these insights open up several practical application directions that address long-standing challenges regarding recyclable thermosets, including flexible elastomer for pressure-sensitive adhesives, chemically recyclable CFRP for lightweight structural components, and corrosion-resistant, fully biobased protective coating.

## 2. Experimental

### 2.1. Materials and reagents

Suberinic acids (SAs) wet paste (32 wt% in water) was received from the Latvian State Institute of Wood Chemistry, it was freeze-dried before any use. Carbon fiber fabric was purchased from FIBREMAX Ltd. All the chemicals and solvents were purchased from Sigma-Aldrich, Fischer, and VWR and were used as received unless noted.

### 2.2. Experimental procedure

#### 2.2.1. SAs thermosets polymerization

Freeze-dried SAs were polymerized in oven at 190 °C for different time length (10 minutes–48 hours). Aggregates and gas bubbles that appeared were removed at the beginning of polymerization before gelation. After polymerization, thermosets were cooled down to room temperature and removed from silicon mold. For aluminum coating, freeze-dried SAs was applied on aluminum and spreaded as it melted, then polymerized as described above.

#### 2.2.2. Recycling of SAs thermosets

20 grams of 4 hours polymerized SAs thermoset was cut into small pieces, then dissolved in boiling 2 M NaOH solution for 12 hours. The resulting solution were precipitated by acidification with 2 M HCl solution until pH 1, the precipitates were washed with DI water until pH reach neutral. The obtained wet paste was freeze-dried and weighed. Yield was calculated according to the following equation: Yield (%) = *W*_2_/*W*_1_ × 100%. *W*_1_ and *W*_2_ represent the sample dry mass before and after the recycling, respectively. The obtained freeze-dried recycled SAs was then polymerized for 3 or 5 hours. Samples were collected after freeze-drying and polymerization for analysis. The rest were recycled in the way described above, in total of 5 times recycling was performed.

#### 2.2.3. Preparation of CFRP with SAs and the recycling

CFRP is prepared with 20 grams of SAs and 2.5 grams of single-ply carbon fiber fabric. SAs was pre-polymerized at 190 °C for 2 hours, aggregates and gas bubbles were removed at the beginning of polymerization before gelation. Then carbon fiber fabric is added to the prepolymerized SAs for further curing of 10 hours. The recycling of the composite was carried out in the same manner as SAs thermosets, 5 grams of the composite was cut to small pieces and refluxed in 100 ml of 2 M NaOH solution overnight, then carbon fiber is separated from the dissolved SAs for washing and drying, the remaining SAs solution is precipitated by acidification with about 100 ml of 2 M HCl solution. The precipitated SAs is then washed and freeze-dried.

### 2.3. Materials characterization

Differential scanning calorimetry (DSC) was performed with Netzsch DSC 214 Polyma with N_2_ as the purge gas (50 mL min^−1^) and using a heating rate of 10 °C min^−1^ in the 25–250 °C temperature range. Calibration was performed using an indium standard for heat flow calibration and zinc standard for temperature calibration. FTIR-ATR was measured with Varian 610-IR FT-IR spectrometer. The (IR) absorbance of samples was measured using an attenuated total reflection-Fourier transform infrared spectroscopic (ATR-FTIR) in the range of 450–4000 cm^−1^. The spectra were baseline corrected using a linear baseline connecting spectral values at around 3900, 2400, 1900, and 1530 cm^−1^, then normalized to the CH_2_ band at around 1465 cm^−1^. NMR spectra were recorded on a Bruker Avance Neo 400 MHz spectrometer (Bruker BioSpin GmbH) that operates at 400.20 MHz for ^1^H nucleus, 160.00 MHz for ^31^P nucleus and 100.63 MHz for ^13^C nucleus. Specific sample preparation and acquisition parameters for each experiment are given below. Sample preparation and acquisition of quantitative ^31^P NMR spectra were carried out following the methodology described in the literature.^13^ Each sample was analysed by duplicate and the results are expressed as the average values. ^13^C NMR was performed by dissolving 100 mg of sample in 1 mL of DMSO_*d_6_* and transferred to a 5 mm NMR tube. Spectra was acquired by using the zgpg30 pulse program and 6500 scans. The mechanical properties of the SAs thermosets were measured using an Instron 5960 universal testing machine (Instron, USA) equipped with a 1 kN load cell at a strain rate of 1 mm min^−1^. The mechanical measurements were performed on rectangular-shaped specimens with dimensions of 100 mm × 10 mm using a gauge length of 10 mm, at least 3 measurements were carried out for each sample. The specimens were conditioned 24 h prior to the measurement and measured at 50% relative humidity (RH) and 25 °C. Dynamic mechanical properties were measured using a dynamic mechanical analyzer (DMA850) in the tension mode. The sample with dimensions of 6 mm × 5 mm × 0.5 mm was scanned from −50 to 250 °C at a heating rate of 3 °C min^−1^. The amplitude was set at 10 μm, and the frequency was 1 Hz. Gel Content, pre-weighed SAs thermosets (about 50 mg) were extracted with a solvent mixture of tetrahydrofuran/ethanol (1 : 1, v/v) (20 mL) for 24 h at room temperature. The insoluble fraction was then dried at 50 °C until a constant weight was reached. The gel content was calculated according to the following equation, gel content (%) = ((*W*_1_ − *W*_2_))/*W*_1_ × 100%. *W*_1_ and *W*_2_ represent the sample dry mass before and after the extraction, respectively. Water contact angle was measured with KRÜSS instruments Drop Shape Analyzer – DSA25, 2 μL water drop was used. At least 3 measurements were carried out for each sample. All the images were processed with the ADVANCE software. The reported data is based on the average contact angle of 3 individual measurement. Water uptake measurement, pre-weighed SAs thermosets (about 50 mg) were immersed in 10 ml DI water for 24 hours, then the weight of SAs thermosets was measured again. The water uptake value was calculated according to the following equation, water uptake (%) = ((*W*_2_ − *W*_1_))/*W*_1_ × 100%. *W*_1_ and *W*_2_ represent the sample mass before and after 24 hours water immersing, respectively. Thermosets swelling in solvents, 3 hours cured SAs thermosets were cut to 1 cm × 1 cm size, then left in 10 ml of water, ethanol, acetone, toluene, and tetrahydrofuran to swell overnight, pictures were taken right after. Anti-corrosion test with electrochemical work station, potentiodynamic polarization tests were performed on bare Al and Al-coated with 24 hours cured SAs thermoset specimens. These electrochemical experiments were performed using a Gamry interface 1010T potentiostat and a three-electrodes glass cell, which consists of the coated/non-coated aluminum specimen as working electrode (area: 4.5 cm^2^), an Ag/AgCl (3 M KCl) as the reference electrode, and a platinum wire as the counter electrode. Potentiodynamic polarization curves were obtained by scanning the electrode potential at a scan rate of 0.167 mV s^−1^ in a NaCl 5% electrolyte at 25 °C, samples were immersed in the electrolyte 24 hours before measurement. Peeling test of pressure sensitive adhesive, peeling test was performed using an Instron 5960 universal testing machine (Instron, USA) equipped with a 1 kN load cell at a strain rate of 300 mm min^−1^ and peel angle of around 90°. The 3-hour cured SAs thermoset (2.5 cm × 10 cm) was conditioned for 24 hours at 50% relative humidity (RH) and 25 °C, then it was evenly coated on a smooth glass slide or stainless steel with a constant pressure provided by the same person. The measurement is carried out within 5 minutes after adhering the thermoset to the substrate, peel force was recorded as a function of displacement. The resistance of paint coatings to separate from substrates was tested according to ASTM D335941.^14^ The multi-blade tool ZCC 2087 was used in this test. The second cut goes across the first one at 90° angle. Then the coated aluminum was lightly brushed to remove loose particles. A 75 mm long tape piece was put in parallel to one of the cuts over the crosshatch pattern, smoothed and firmly rubbed with finger/fingernail. In the end, the tape steadily removed for 0.5 to 1.0 s at an angle which was close to 60° to the substrate. The result was evaluated based on the extent of coating removal after the test.

## 3. Results and discussion

### 3.1. Controlling thermoset network formation through polymerization kinetics

**Fig. 1:**
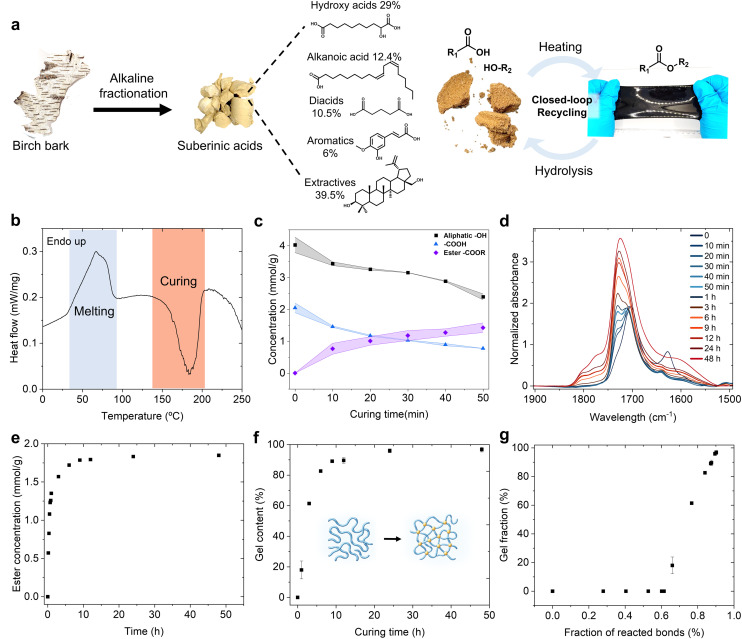
Catalyst-free thermal polycondensation of SAs. (a) Schematic of the thermoset preparation from SAs, and the reversible ester network enabled recycling (the shown composition of SAs includes the percentage of each category of compounds and their representative structures. For a detailed list of compounds see Table S1. (b) DSC trace of SAs showing melting and curing regions. (c) Quantitative analysis of ester formation in the first 50 minutes of polymerization. (d) IR spectra of SAs from 0 to 48 hours of polymerization in the carbonyl region. (e) Estimated ester group growth after extended curing time (all data points are shown). (f) Gel content of the thermosets. (g) Correlation between gel fraction and fraction of reacted bonds. The upper and lower boundary of the shaded area behind the data points in (c) represent the two individual replicates making up the mean. Error bars in (f) and (g) represent one standard deviation from the mean values (*n* = 3).

Our exploratory work showed that variations in curing time and temperature lead to distinct material properties, underscoring the importance of precise thermal control to achieve targeted performance. In the dry state, SAs appear as solidified wax that undergoes a broad melting transition between 30 and 90 °C, as indicated in the endothermal DSC peak (Fig. 1b). This molten state enables efficient mixing and processing before crosslinking network formation. Upon further heating, polymerization is initiated within a well-defined exothermal region of approximately 130–205 °C. Within this range, polycondensation between the abundant hydroxyl and carboxylic acid groups becomes the dominant reaction pathway, progressively establishing the crosslinked polyester structure (Fig. 1a and b). Quantitative ^31^P NMR spectroscopy measurements revealed a significant decrease in aliphatic hydroxyl content from 4.0 mmol g^−1^ to 2.4 mmol g^−1^ within the first 50 minutes of curing (Fig. 1c and Fig. S1–4). Simultaneously, the concentration of carboxylic acid groups declined from 2.0 mmol g^−1^ to 0.8 mmol g^−1^, consistent with ester bond formation as shown in the IR spectra (Fig. 1d and Fig. S5), where there is a distinct shift in the carbonyl stretching frequency from 1702 cm^−1^ (carboxylic acid) to 1727 cm^−1^ (ester). A closer look at the conversion profiles (Fig. S6) reveals a clear structure–reactivity relationship. The primary aliphatic hydroxyls (C28-OH in betulin) react rapidly, reaching 60% conversion at 60 minutes, while the secondary C3-OH only attains 30% conversion, consistent with the steric congestion of secondary alcohols. Phenolics from condensed guaiacyl units also react slowly (below 30%), reflecting their inherent steric constraints. The behaviour of carboxyl groups highlights a similar trend, fatty acids –COOH react extensively (70%), while the bulky triterpenoid –COOH merely reaches a conversion of around 40% at the end. Hydroxyacid-derived –OH groups reach 50% conversion at the end, contributing strongly to early-stage network formation. Notably, the disproportionate reduction in hydroxyl content suggests that excess hydroxyl groups may undergo oxidation, potentially involving radical species formed from thermally oxidated non-conjugated C=C bonds,^15^ contributing to the evolving crosslinked network beyond the main esterification.

After approximately 50 minutes of curing at 190 °C, the system reaches the gelation point where the molten SAs transform into a more viscous crosslinked gel with limited solubility due to the formation of an infinite network, which prevents the reaction from being followed by ^31^P NMR. To quantify curing beyond this stage, a calibration curve is built based on pre-gelation ^31^P NMR and FTIR data (Fig. S7), enabling time-resolved analysis of the curing process and elucidation of dominant reaction pathways at different stages. This analysis shows that the formation of new ester bonds slows down sharply after 1 hour and gradually reaches a plateau after 48 hours of extended curing (Fig. 1e). Due to the limited movement of reactive sites by the crosslinks and much lower reactant concentrations, unreacted functional groups become less accessible to each other. Eventually, the secondary oxidation becomes more pronounced, as evidenced by an increase in the peak near 1776 cm^−1^ in the IR spectra, which could be attributed to the formation of γ-lactones. The unsaturation in γ-lactone is known to cause carbonyl stretching peak shifts to 1773 cm^−1^–1780 cm^−1^ region.^15^

Consistent with the increasing formation of ester bonds, the gel content of the thermosets rises rapidly during the early curing stage, reaching 80% after six hours, and gradually increases to 96.7% after 48 hours of extended curing (Fig. 1f). The estimated gelation point occurs when 61.4% of the ester-forming groups have reacted (Fig. 1g). By applying this critical conversion to the Flory–Stockmayer gelation model, the number average functionality of each monomer is estimated to be 2.67 (calculated with eqn (S1)), suggesting that the SAs comprise a mixture of di- or tri-functional monomers. This result reflects the known composition of hydrolyzed suberin, which contains hydroxyl diacids, hydroxyl acids, and diacids, as reported in previous studies.^9,16^ The 96.7% gel content is comparable to that of trifunctional or even higher functional plant oil-based epoxy resins.^17,18^ Commonly used epoxy resin like diglycidyl ether of bisphenol A (DGEBA) with diamine are difunctional, while higher functionality lowers the gel point and increases the crosslinking density.^19^

### 3.2. Tunable material properties

**Fig. 2:**
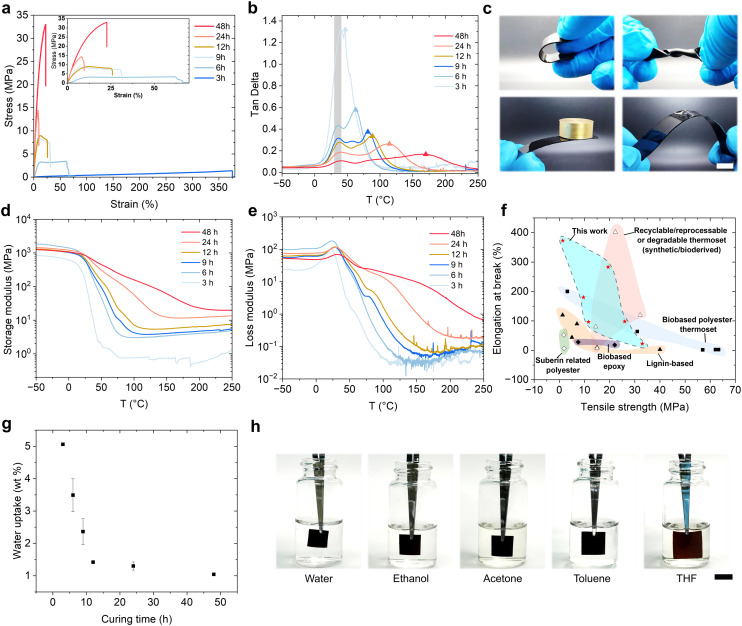
Mechanical performance of SAs thermosets and their water and solvent stability. (a) Representative stress–strain curves from tensile tests of thermosets cured for different hours. (b) Tan delta-temperature curve of thermosets cured for different hours. (c) Appearance of elastic 3 hours cured sample (upper) and rigid 48 hours cured sample (lower, weight of the load: 22.5 grams). (d and e) The effect of temperature on the storage modulus and loss modulus of thermosets cured for different hours (3–48 h). (f) Mechanical properties of SAs thermosets (present work) compared with other biobased thermosets and recyclable,^11,12,21–29^ reprocessable or degradable thermosets (values used in this graph can be found in Table S2). (g) Influence of curing time on 24-hour water uptake of thermosets. The error bars represent one standard deviation from the mean (*n* = 2). (h) Swelling of thermosets (3 hours cured) after immersion in different solvents for 24 hours. Scale bar: 1 cm.

The thermosets exhibit distinct mechanical responses depending on the fraction of reacted bonds, suggesting a potentially different range of applications. From three hours to 48 hours of curing, the ultimate tensile strength of SAs thermosets increases from 1.4 MPa to 32.9 MPa, consistent with the rising fraction of reacted bonds from 76% to 90% (Fig. 2a, 1e and g). Correspondingly, the elongation at break decreases from 375% to 22%, which represents the transition from a flexible elastomer to a rigid, highly crosslinked thermoset (Fig. 1g, 2a, c and Fig. S8). These mechanical properties achieved in the present SAs thermosets are comparable to other types of biobased thermosets, such as those derived from lignin and epoxies, and they outperform previously reported suberin- based thermosets (Fig. 2f and Table S2). Furthermore, SAs based thermosets match the mechanical performance of state-of-the-art synthetic and bioderived recyclable thermosets, while offering a genuinely straightforward polymerization process without additional crosslinkers, catalysts, or chemical modifications. This combination of high performance and formulation simplicity positions suberinic acids as a compelling and scalable building blocks for fully biobased thermosets. Dynamic mechanical analysis (DMA) reveals two characteristic relaxation processes (Fig. 2b). The first relaxation appears at a lower temperature (∼36 °C) due to the presence of unreacted or partially reacted fatty acid monomers, which become progressively less prominent as the degree of polymerization increases (Fig. 2b). The second relaxation at higher temperatures corresponds to the true glass transition of the bulk crosslinked structure, which shifts markedly from 46 °C to 170 °C when the curing time increases from three hours to 48 hours, reflecting the enhanced network rigidity and reduced chain mobility associated with a higher crosslinking density (Fig. 2b). At elevated temperature, the storage modulus of highly crosslinked thermosets does not exhibit a pronounced decrease but instead reaches a rubbery plateau (Fig. 2d). This behavior is attributed to the extensive covalent crosslinking, which restricts molecular chain movement and prevents the network from undergoing significant viscoelastic softening.^20^ Similarly, the loss modulus of more cured thermosets exhibits a broader and less intense peak compared to that of lightly crosslinked ones (Fig. 2e).

**Fig. 3:**
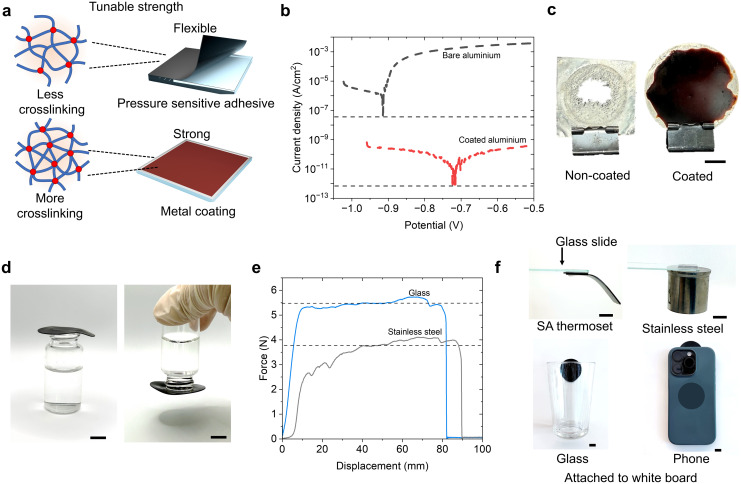
Tunable mechanical properties enable diverse applications of the suberinic acid thermosets. (a) Comparison of cross-linking density in relation to flexibility and rigidness of the material. (b) Potentiodynamic polarization curves of coated (24 h cured, red line) and reference bare aluminum substrate (black line). (c) Digital photograph of bare aluminum and SAs thermoset coated aluminum panel after the anti-corrosion test. (d) A glass vial with water inside, closed by an elastic 3 h cured SAs thermoset. (e) Result of peeling test on glass and steel. (f) Digital photographs showing 3 h cured sample adhere to different substrate. Scale bars (1 cm).

Fatty acids are in general hydrophobic due to the non-polar hydrocarbon chains that tend to aggregate together to minimize their contact with water through the hydrophobic effect. However, the covalently crosslinked SAs become more hydrophilic as the number of crosslinks increases (Fig. S9). This is likely because the crosslinked fatty acids’ alkanes become topologically more constrained by the covalent network and therefore result in a weaker hydrophobic effect compared with the more freely mobile fatty acids.^30^ In addition, prolonged thermal exposure promotes partial oxidation of SAs, leading to the formation of additional oxygen-containing polar functional groups, as seen by the emergence of new bands in the 1750–1825 cm^−1^ region (Fig. 1d). These bands may be attributed to carbonyl-containing oxidation products, like γ-lactone and related species. The increase of these polar functionalities likely increases the surface hydrophilicity. A similar phenomenon has been reported regarding the ageing of alkyd paints.^31^ On the other hand, the more extensively crosslinked SAs thermosets absorb less water, with the water content decreasing from 5 wt% for the 3-hour-cured sample to 1 wt% for the 48-hour-cured sample (Fig. 2g). This result reveals that distinct mechanisms drive surface wetting and bulk material water uptake. A highly crosslinked network limits the water diffusion through the material and therefore absorbs less water.^32^ The swelling of SAs thermoset in water (5.6%) is limited due to the low water absorption ratio. In organic solvents including ethanol (15%), acetone (14%), and toluene (57%), swelling is more substantial, and it reaches a maximum in tetrahydrofuran (THF) (190%) as shown in Fig. 2h and Fig. S10. These tunable material properties extend the use of such material from less crosslinked flexible pressure-sensitive adhesive to highly crosslinked strong metal coating (Fig. 3a).

Motivated by the low water uptake and high strength of highly crosslinked SAs thermosets, we anticipate that such a material could function as a durable protective coating that remains stable in a corrosive 5% sodium chloride aqueous environment (neutral pH). Such a condition is particularly aggressive towards aluminium, as chloride ions readily breaks down the naturally formed passivation (Al_2_O_3_) and therefore induces localized pitting and accelerates dissolution. To test whether the SAs thermoset can suppress this process by restricting water and ion penetration, aluminum substrate was coated with SAs and cured for 24 hours, followed by corrosion evaluation by measuring the corrosion current density (CCD) using a potentiodynamic polarization scan in a standard electrochemical setup. The coated sample exhibited a five-order-of-magnitude reduction in CCD, decreasing from 7.5 × 10^−8^ to 8.3 × 10^−13^ A cm^−2^ (Fig. 3b), indicating significantly enhanced corrosion protection, which outperforms most other reported biobased metal coatings in terms of inhibition efficiency (Table S3).^33,34^ Visual inspection after the corrosion test further confirmed this protection, the uncoated aluminum strip exhibited severe pitting and surface degradation characteristic of chloride induced corrosion.^35^ In contrast, the sample coated with 24 hours cured SAs thermoset remained intact with no visible damage or surface alteration (Fig. 3c). This stark difference highlights the ability of the SAs thermosets to block the primary corrosion pathways in chloride-containing environments by limiting both water penetration and chloride ingress, thereby preserving the integrity of the underlying metal.

Moreover, the shift of corrosion potential in the noble direction further indicates the coating's protection against corrosion (−0.91 V to −0.71 V *vs.* Ag/AgCl for non-coated Al and Al coated with 24 hours cured SAs thermoset). The present SAs thermoset coating show inhibition efficiency of nearly 100%. For comparison, lignin-based coatings typically exhibit an inhibition efficiency of 98–99% under similar conditions.^4,36^ Similarly, the advanced hydrophobic composite coatings, such as PDMS + MXene, can achieve nearly 100% protection. Commercial epoxy coatings show inhibition efficiency up to 98.5% under comparable conditions.^37^ The SAs coatings developed in this work demonstrate outstanding performance among state-of-the-art biobased and petroleum-based coatings. This excellent performance is likely attributed to the intrinsic water resistance of SAs coating, limiting the interaction between metal surface and corrosive media. Additionally, SAs coating offers distinct advantage over conventional systems owing to its fully biobased origin, simplicity of preparation, and recyclability. Another critical factor influencing long-term performance is the adhesion of the coating on the substrate. Strong adhesion prevents premature failure due to delamination and helps maintain the coating's protective function. The cross-cut adhesion test confirmed excellent adhesion, showing no visible detachment in the cut area and smooth edges around the incisions (Fig. S11).

The coating achieved the highest rating of 5B (on a scale from 5B to 0B), indicating minimal to no detachment. This strong adhesion is likely facilitated by multiple factors. Firstly, the polar ester groups within SAs thermosets can interact favourably with the hydrophilic metal oxide surface. The low viscosity of molten SAs before crosslinking ensures effective wetting and infiltration into microscale surface asperities.

In the end, the progressive crosslinking consolidates these contacts, generating a partially interpenetrated and mechanically interlocked interfacial layer. Together, these effects endow the coating with exceptional interfacial stability under long-term exposure, thereby preserving its anticorrosive functionality.

### 3.3. Recycling and reprocessing

One of the most persistent challenges of conventional thermosets is their intrinsic limited recyclability and reprocessability. The permanent crosslinked network that provides dimensional stability simultaneously prevents remelting or reshaping, resulting in materials that are difficult to repurpose at end of life. To address this issue, many emerging studies have developed various recycling approaches, including the incorporation of cleavable segments, methanolysis, and acetolysis, as well as the development of covalent adaptable networks and vitrimers.^38–41^ While these strategies demonstrate promising progress, many require specialized monomers, catalysts, or complex reaction conditions, which potentially limit their scalability.

**Fig. 4:**
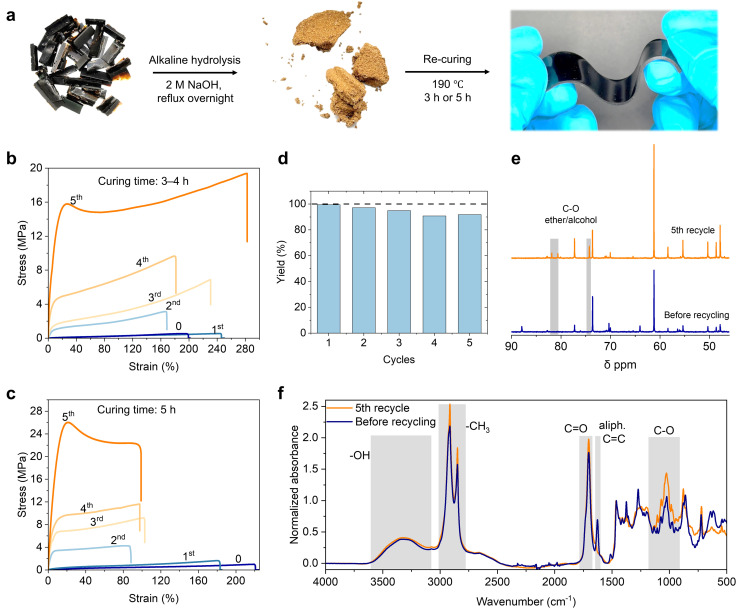
Multi-cycle recycling and reprocessing of SAs thermosets. (a) Schematic illustration of recycling and reprocessing of SAs thermosets. (b) Representative stress–strain curves of SAs thermosets before and after 1–5^th^ recycling (reprocessed by curing for 3 hours). (c) Representative stress–strain curves of SAs thermosets before and after 1–5^th^ recycling (reprocessed by curing for 5 hours). (d) Materials recovery yield from each cycle of recycling (all data points are shown). (e) ^13^C NMR spectra of SAs before recycling and after the 5th recycle. (f) IR spectra of SAs before recycling and after the 5th recycle.

In contrast, here we explored a straightforward chemical recycling of the SAs thermosets through alkaline hydrolysis, followed by acid precipitation, and thermal polycondensation to recover a thermoset structure (Fig. 4a). This approach demonstrates high-yield multi-cycle reprocessability, with five recycling rounds showing a yield of above 90% from each cycle without any compromise in the mechanical performance of reprocessed thermosets (Fig. 4b–d, Fig. S12 and 13). Remarkably, there is a trend that the mechanical performance of recycled SAs thermoset increases after each cycle. After five times of recycling, recycled SAs thermosets cured for three hours exhibited an increase in ultimate tensile strength from 0.5 MPa to 19.3 MPa, accompanied by an increase in ultimate tensile strain from 200% to 280% (Fig. 4b, Fig. S12 and 13). The elasticity of the thermosets is maintained mainly for those that have been repolymerized for three hours. The five-hour-repolymerized thermosets show an enhanced ultimate tensile strength but also exhibit reduced elongation at break, with the tensile strength increasing from 1 MPa to 26 MPa and the strain decreasing from 220% to 100% (Fig. 4c, Fig. S12 and 13).

Together, these findings demonstrate that SAs thermosets not only support closed-loop chemical recycling but can even achieve significantly enhanced mechanical performance with repeated reuse, distinguishing them from most recyclable thermoset systems where degradation, dilution, or crosslink scission typically reduce material properties over time. This enhancement in strength may be attributed to the oxidation of SAs during recycling, leading to the formation of additional oxygen-containing functional groups (Fig. 4e and f),^15,42^ In support of this hypothesis, FTIR and quantitative ^31^P NMR analyses show an increase in carboxylic acid groups content after repeated depolymerization and repolymerization (Fig. 4f and Table S4). These additional –COOH groups enables the formation of more ester linkages during repolymerization, resulting in higher crosslinking density and thus improved mechanical strength. This combination of simplicity, high yield, and property enhancement positions SAs based thermosets as a promising platform for scalable, fully biobased circular materials. However, scaling up presents challenges regarding heat transfer and the polymer homogeneity, as reflected by the variation in materials performance observed in the present study, due to the sample size differences between the thermosets shown in Fig. 2a (2 g) and Fig. 4b and c (20 g), the elongation at break decreased from 370% (3 h curing) to 200% (4 h curing), despite the longer curing time applied to the larger sample. Notably, a curing time of 3 h was insufficient for the 20 g sample to form a stable gel, indicating slower network formation in the larger volume of material. This behavior likely arises from the reduced heat transfer efficiency and less uniform curing throughout the larger sample, which influences the crosslinking density and therefore the mechanical properties. It is therefore more relevant to compare the materials based on their crosslink densities rather than curing time alone.

**Fig. 5:**
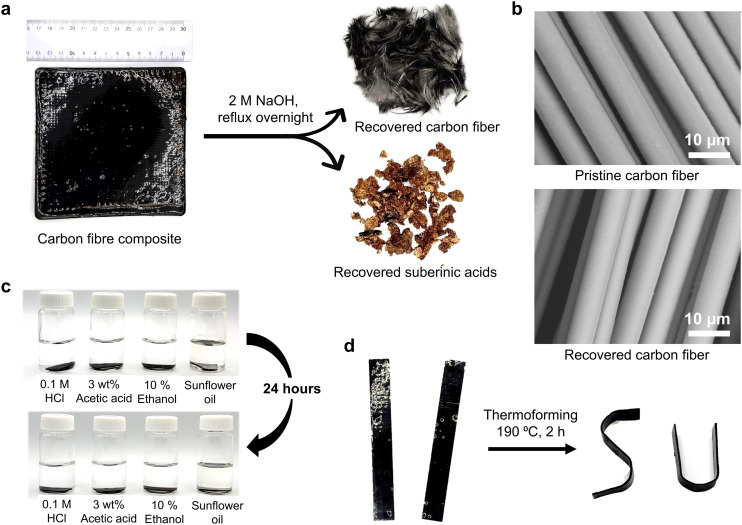
Recyclable SAs-based carbon fibre reinforced composite. (a) Representative photograph of the composite and its components, with carbon fibers and SAs recovered by alkaline hydrolysis. (b) SEM images comparing pristine and recovered carbon fibers, showing unchanged structural integrity. (c) Chemical stability of the composites after 24-hour immersion in 0.1 M HCl, 3 wt% acetic acid, 10% ethanol, and sunflower oil. (d) Thermoforming of the composites at elevated temperature.

The successful recycling of the thermosets allows for their use as a polymer matrix in recyclable carbon fiber reinforced composites (Fig. 5a), offering a potentially sustainable alternative to conventional non-recyclable epoxy resin–based carbon fiber composites. The resulting SAs/single-ply carbon fiber composite exhibited an ultimate tensile strength of 148 MPa (Fig. S14). Alkaline hydrolysis enabled the efficient separation of carbon fibers (recovery yield: 98.6%) and SAs, without compromising the structural integrity of the carbon fibers as confirmed by the SEM and FTIR analyses (Fig. 5b and Fig. S15). The recovered SAs can be reused for manufacturing new thermosets or composites, further enhancing material circularity. To evaluate the chemical stability of the composites, samples were immersed in 0.1 M hydrochloric acid, 3 wt% acetic acid, 10% ethanol, and sunflower oil for 24 hours, and their weights were recorded before and after exposure. All samples demonstrated excellent stability, with negligible changes in weight and appearance (Fig. 5c and Fig. S16). Furthermore, the composites could be thermoformed at 190 °C, enabling reprocessing into new geometries even after polymerization (Fig. 5d).

## 4. Conclusions

This work demonstrates the feasibility of producing high-performance, recyclable, and upcyclable thermosets entirely from birch outer bark, a forestry by-product. The successful synthesis, recycling and upcycling of suberinic acid thermosets and their carbon fiber composites effectively extends the lifetime of these fully biobased materials, marking a significant step toward circular thermoset materials. By tuning the degree of crosslinking, we obtained materials with tunable mechanical properties, enabling their use across diverse applications. The stiffer materials, with a crosslinking degree close to 90%, are suitable for use in metal coatings and carbon fiber composites, whereas the more elastic materials, with a lower crosslinking degree of around 76%, are better suited for applications such as watertight sealing materials and pressure-sensitive adhesives. Conventional thermosets are typically synthesized from petroleum feedstocks that might involve toxic curing agents such as formaldehyde, bisphenol A, or anhydrides, which raise concerns regarding environmental persistence and potential risks to human health during production, use, and disposal.^43^ In contrast, the present fatty acid–based thermosets avoid all of these hazardous precursors, offering a safer and potentially more sustainable alternative.

Closed-loop chemical recycling not only ensures high material recovery yields but also leads to enhanced mechanical performance after each cycle. This capability enables the production of recyclable carbon fiber reinforced composites with a fully biobased polymer matrix, where the matrix and carbon fibers can be efficiently separated without compromising fiber integrity. Because no fossil-derived or toxic small chemicals are used in the synthesis, the materials present a lower hazard profile during production, use, and end-of-life processing, while their natural origin enhances prospects for biodegradation under appropriate conditions. This approach substantially reduces environmental impacts compared to conventional non-recyclable thermosets and broadens the range of sustainable applications, including coatings, rubbery gaskets, adhesives, and composites, providing a practical and safer pathway toward circular thermoset materials.

## Author contributions

F. W., A. J. H.-A., and M. H. S. conceived the idea and designed the experiments. I. P., A. J. H.-A., and M. H. S. supervised the project. F. W., R. G., P. V., M. A., and A. J. H.-A. performed the experiments. J. R., M. A., I. P., A. J. H.-A., and M. H. S. provided constructive suggestions. F. W., R. G., P. V., I. P., A. J. H.-A., and M. H. S. contributed to data preparation, analysis and manuscript drafting with input from all authors. J. R. supplied the suberinic acids for this study. M. H. S. provided resources for this study. All authors discussed and revised the paper.

## Conflicts of interest

There are no conflicts to declare.

## Supplementary Material

GC-028-D5GC06834G-s001

## Data Availability

Source data for this article is available at Zenodo at https://doi.org/10.5281/zenodo.17961292. Supplementary information (SI) is available. See DOI: https://doi.org/10.1039/d5gc06834g.
