# Supplementary material for: Upcycling birch bark suberin into versatile and recyclable thermosets
Source: Green Chem. 2026 Mar 17;28(19):8147–57. doi: 10.1039/d5gc06834g (PMC13016059; doi:10.1039/d5gc06834g)
Supplement: GC-028-D5GC06834G-s001 [file GC-028-D5GC06834G-s001.pdf]

Supplementary Information (SI) for Green Chemistry.
This journal is © The Royal Society of Chemistry 2026

Supplementary Material

Upcycling birch bark suberin into versatile and recyclable thermosets

Fengyang Wang [1], Ruslan Gryaznov [1,2], Pavel Vostrejs [1], Matilda Andersson [1], Jānis Rižikovs [1], Ievgen Pylypchuk [1], Alberto J. Huertas-Alonso*[1], Mika H. Sipponen*[1,2]

[1] Department of Chemistry, Stockholm University,

SE-10691, Stockholm, Sweden

[2] Department of Chemistry, Wallenberg Wood Science Center, Stockholm University,

SE-10691, Stockholm, Sweden

[3] Latvian State Institute of Wood Chemistry, Biorefinery Laboratory, Latvia

*Corresponding to albertojose.huertasalonso@su.se; mika.sipponen@su.se

Table S1 shows the detailed composition of suberinic acids used in the current study, which was modified from previously published data.

Table. S1. Composition of suberinic acids, modified from previously published data[1].

**Hydroxyacids**	**29.2%**	**Diacids**	**10.4%**
2-hydroxydecanedioic acid	21.3%	pentanedioic acid	5.4%
22-hydroxydocosanoic acid	4.7%	10,12-docosadiynedioic acid	2.4%
20-hydroxyicosanoic acid	2.6%	Hexadecanedioic acid	1.4%
3-hydroxyhexadecanoic acid	0.6%	Octanedioic acid	1.2%
**Extractives**	**39.5%**	**Aromatics**	**6%**
Betulin	36%	isoferulic acid	4.8%
Lupeol	3.5%	Oxyisoflavone	0.9%
**Alkanoic acid**	**12.4%**	Vanilic acid	0.3%
9,12-octadecadienoic acid	8.2%	**2-oleoylglycerol**	**0.8%**
Octanoic acid	4.2%	**Others**	**1.7%**

Figure S1 shows the ^{31}P NMR spectra used for the quantitative analysis of hydroxyl groups in suberinic acids cured for different time lengths.

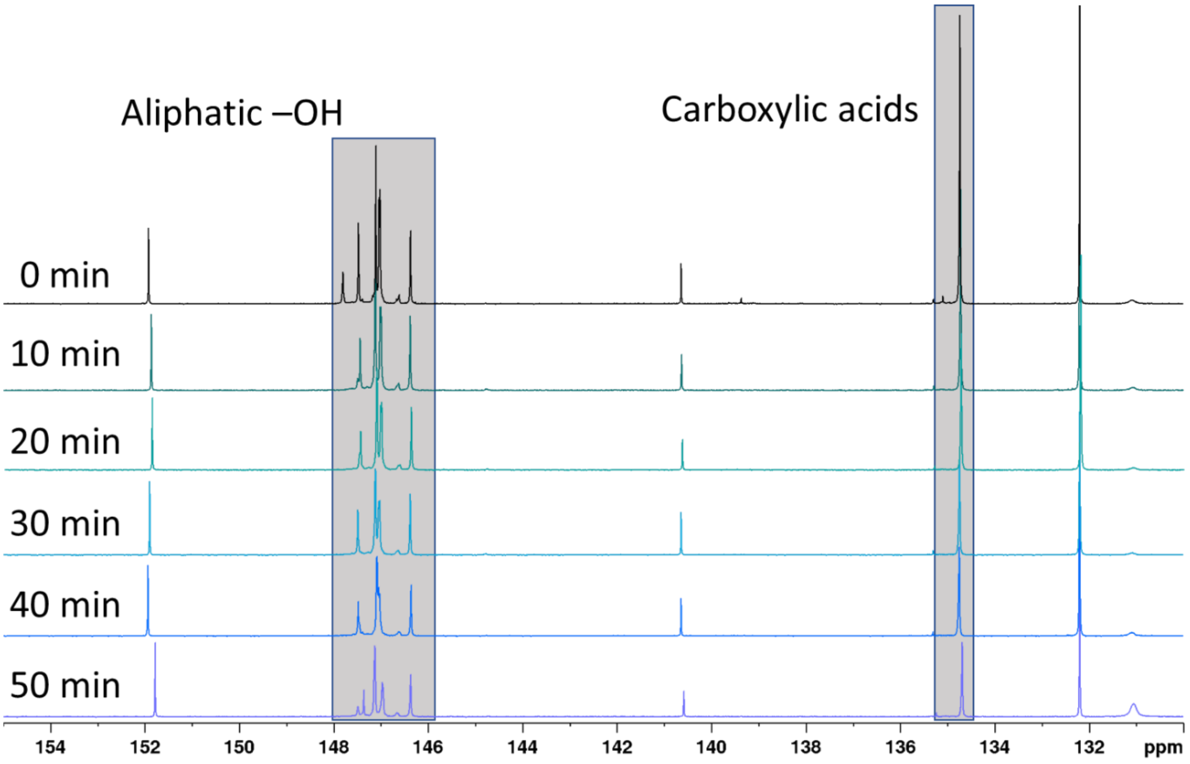

Fig. S1. Comparison of aliphatic -OH region and -COOH region of suberinic acids in ^{31}P NMR spectra from 0 to 50 minutes of thermal polymerization at 190 ºC. Spectra are normalised to internal standard.

Figure S2 highlights the carboxylic acids region in the ^{31}P NMR spectra of suberinic acids. The blue area indicates betulinic acid, red area refers to trans-ferulic acid, and the brown area is from aliphatic carboxylic acids.

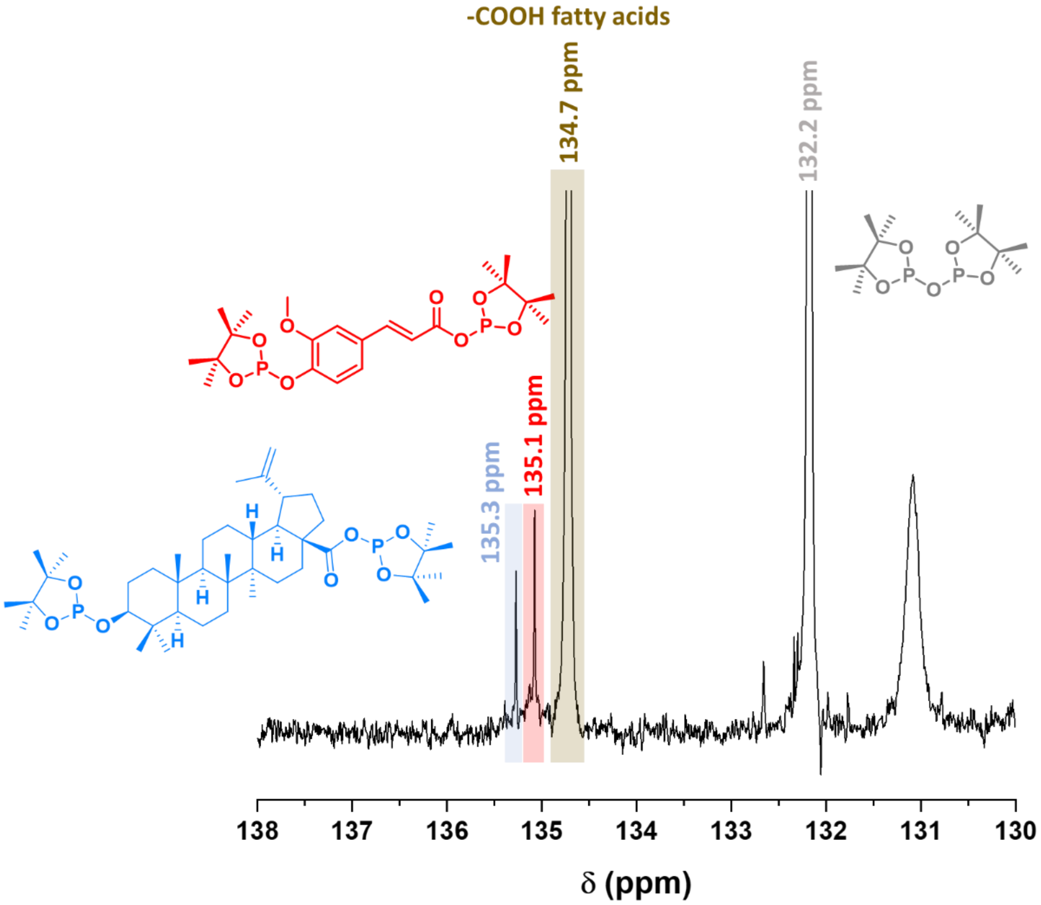

Fig. S2. Carboxylic acids region in the ^{31}P NMR spectra of suberinic acids.

Figure S3 highlights the aliphatic -OH region in the ^{31}P NMR spectra of suberinic acids. The blue area at 147 and 146 ppm represents the primary and secondary -OH of betulin, respectively. The brown area refers to the -OH from hydroxy acids.

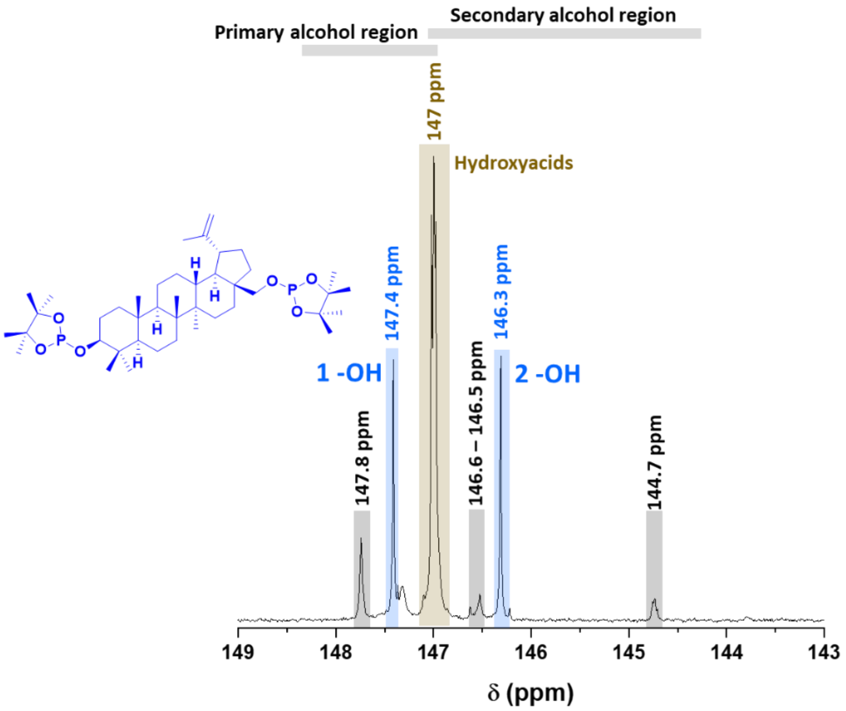

Fig. S3. Aliphatic -OH region in the ^{31}P NMR spectra of suberinic acids.

Figure S4 highlights the aliphatic -OH region in the ^{31}P NMR spectra of suberinic acids. The peaks at 140 and 139 ppm represent the secondary -OH of condensed guaiacyl and ferulic acid, respectively.

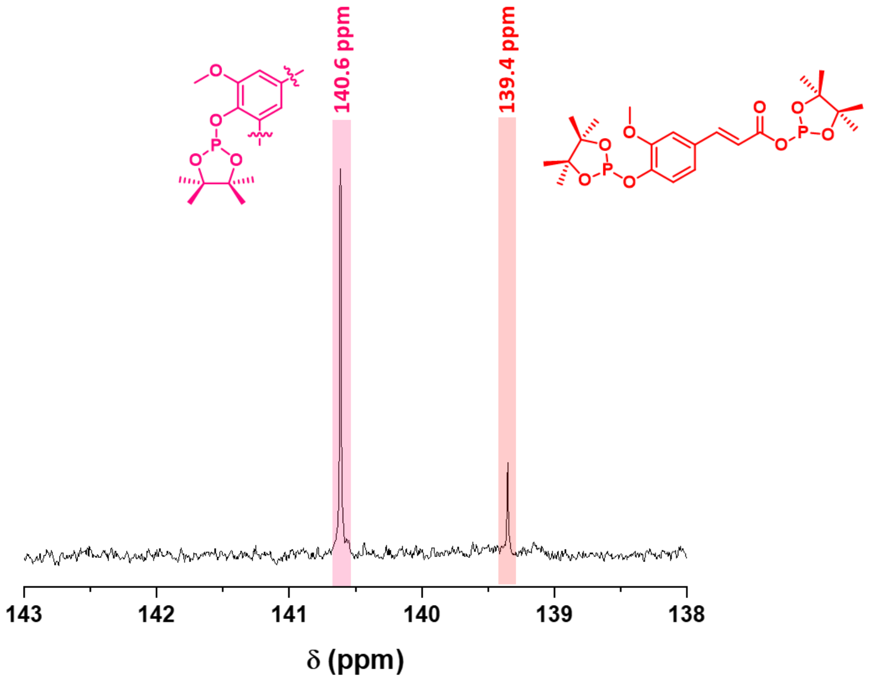

Fig. S4. Phenolic -OH region in the ^{31}P NMR spectra of suberinic acids.

Figure S5 shows the full representative ATR-FTIR spectra of suberinic acids polymerized from 0 to 48 hours at 190 ºC, where intensity of -OH group drops over time and C=O (ester) peak intensity increases.

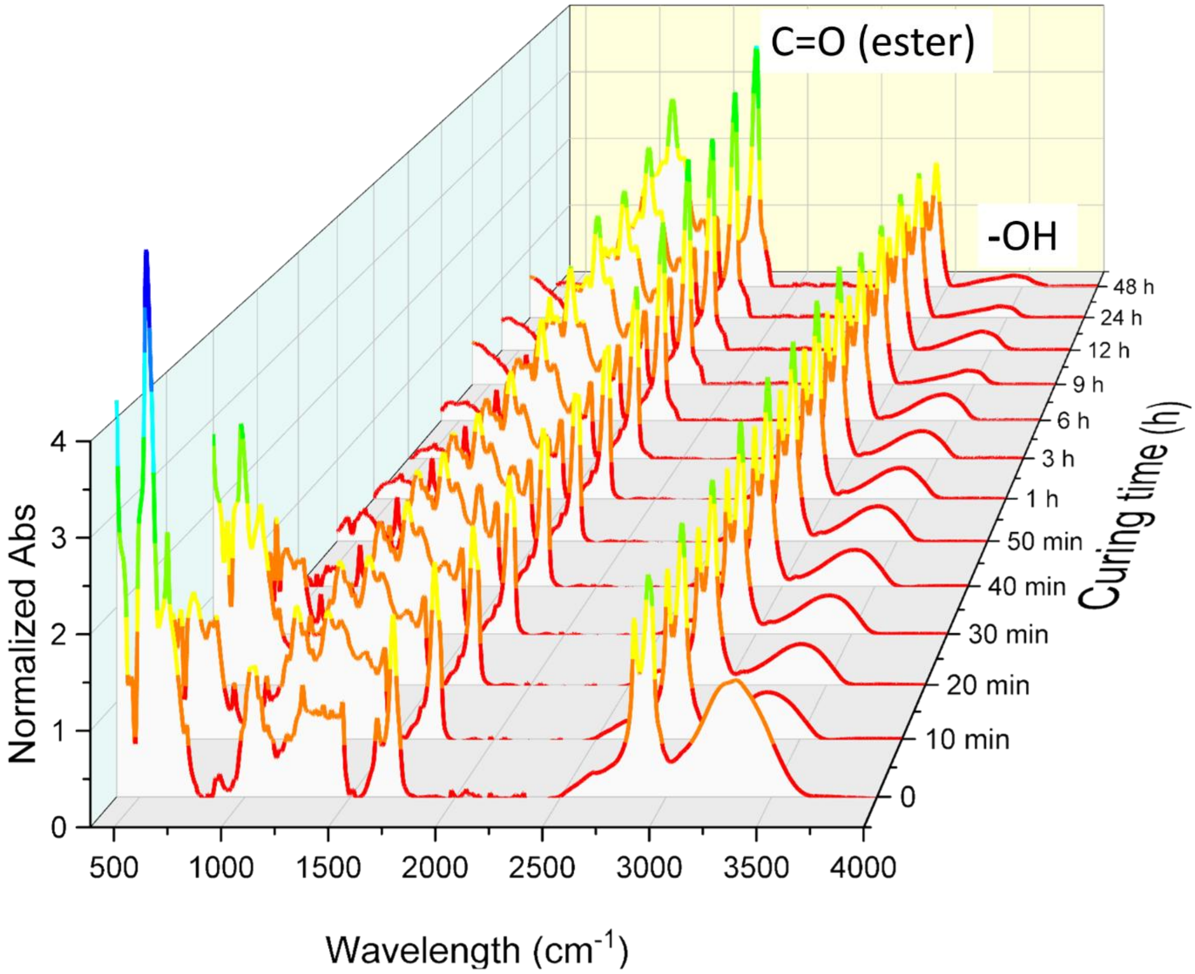

Fig. S5. Full representative ATR-FTIR spectra of suberinic acids polymerized from 0 to 48 hours at 190 ºC.

Figure S6 shows the quantitative analysis of the conversion rates of the main species in suberinic acids involved in the polycondensation reaction.

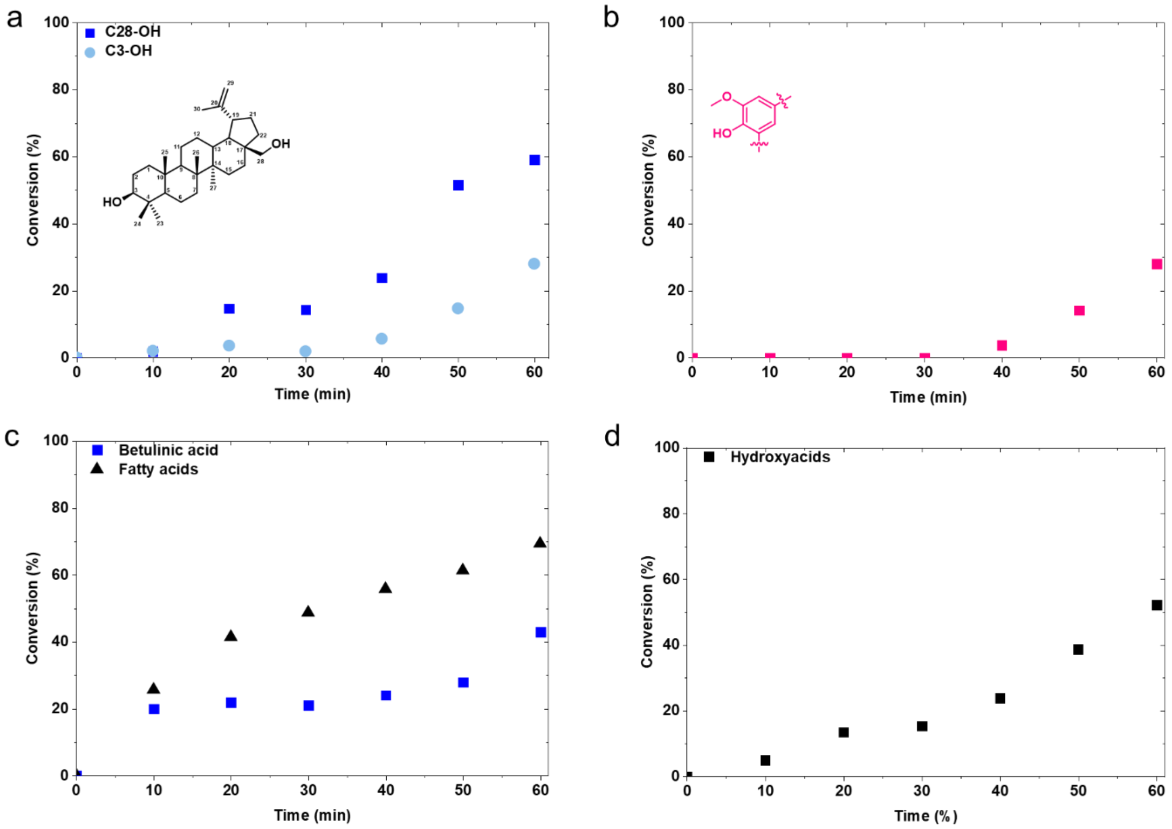

Fig. S6. The conversion rate of the main species in suberinic acids during the first hour of polymerization. a) Conversion rates of primary -OH and secondary -OH in betulin. b) Conversion rate of the secondary -OH from condensed guaiacyl. c) Conversion rates of -COOH from fatty acids and betulinic acid. d) Conversion rate of the -OH from hydroxyacids.

Figure S7 a) shows the change of normalized absorbance at 1727 cm-1 in the IR spectra of suberinic acids polymerized from 0 to 48 hours at 190 ºC. Figure S5 b) shows the correlation between the newly formed ester concentration (recalculated from ^{31}P NMR based on the decrease in carboxylic acids concentration) and normalized absorbance at 1727 cm-1 in IR spectra, as well as the fitting of the correlation.

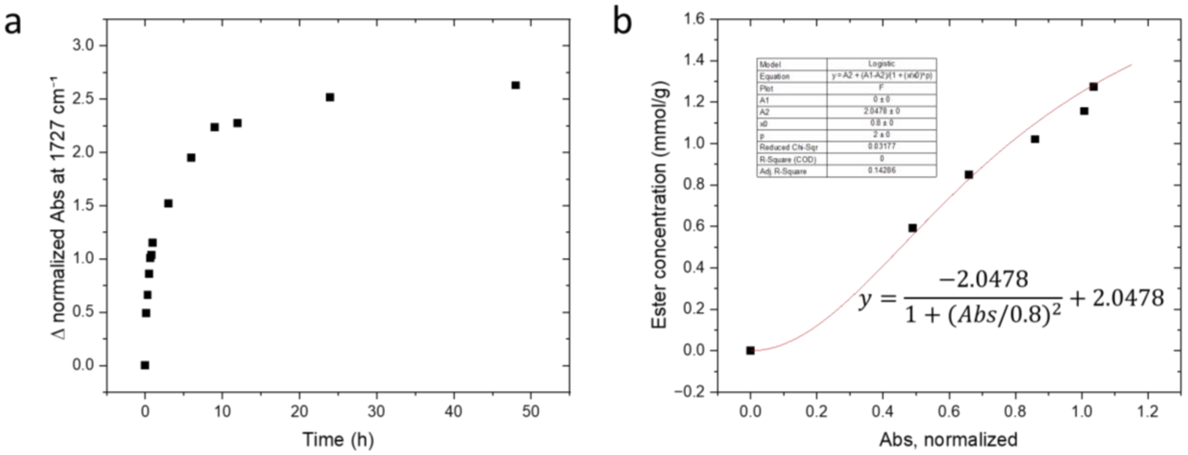

Fig. S7. a) Increase in the normalized absorbance at 1727 cm^{-1} in IR spectra from 0 to 48 hours. b) Fitting of the ester concentration (measured with ^{31}P NMR) and increases in the normalized absorbance at 1727 cm^{-1} in IR spectra.

Equation S1 shows the calculation of the average functionality of suberinic acids from the degree of reaction at gel point.

Equation S1

$$average\ functionality = 1 + \frac{1}{degree\ of\ reaction\ at\ gel\ point}$$

Figure S8 shows the stress-strain curves of suberinic acids thermosets polymerized at 190 ºC for different time lengths (3h, 6h, 9h, 12h, 24h, 48h).

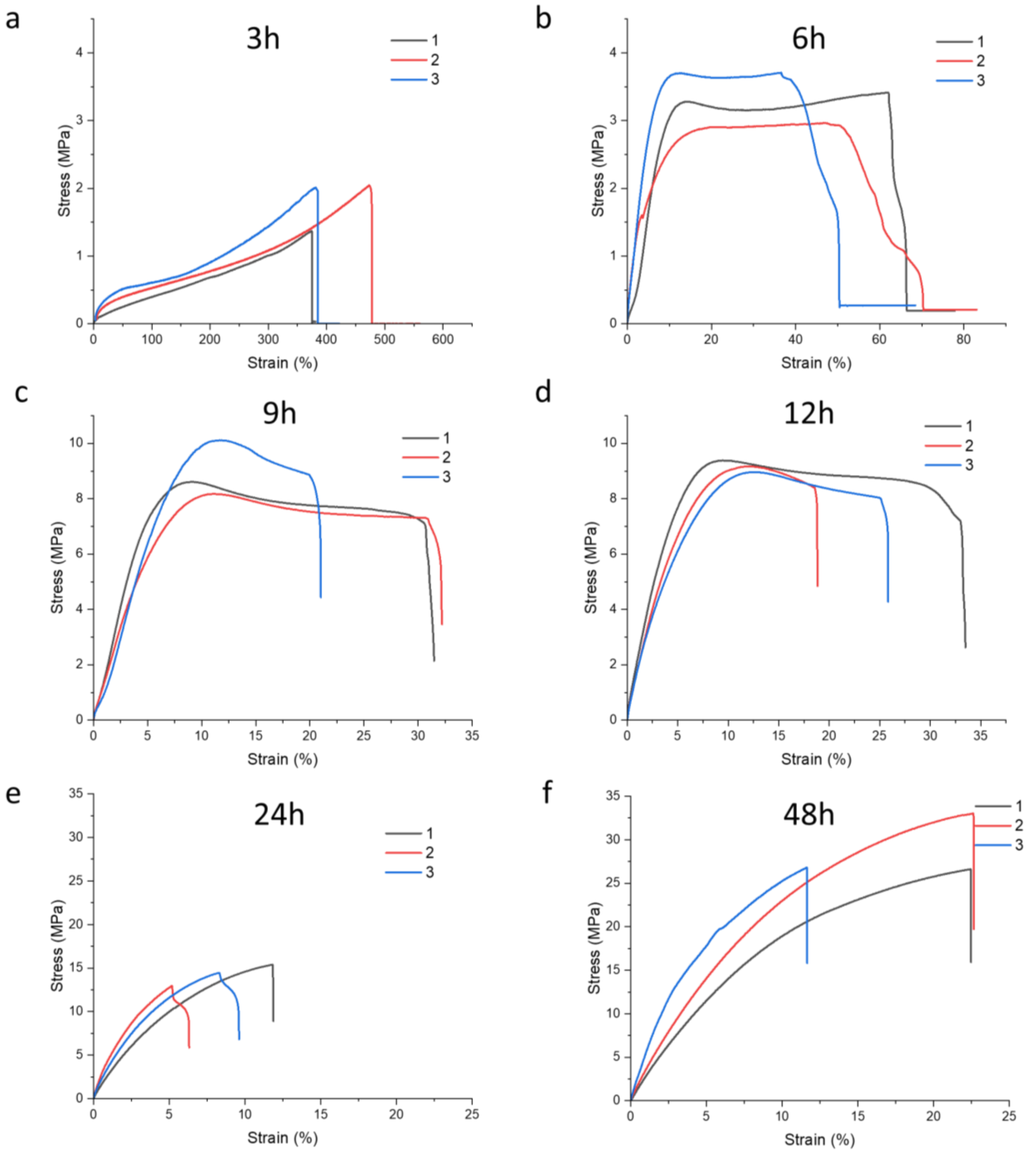

Fig. S8. Stress-strain curves of suberinic acids thermosets polymerized at 190 ºC for different time lengths. a) 3 hours, b) 6 hours, c) 9 hours, d) 12 hours, e) 24 hours, and f) 48 hours.

Figure S9 shows the decrease in water contact angles of suberinic acids thermosets with increasing polymerization time.

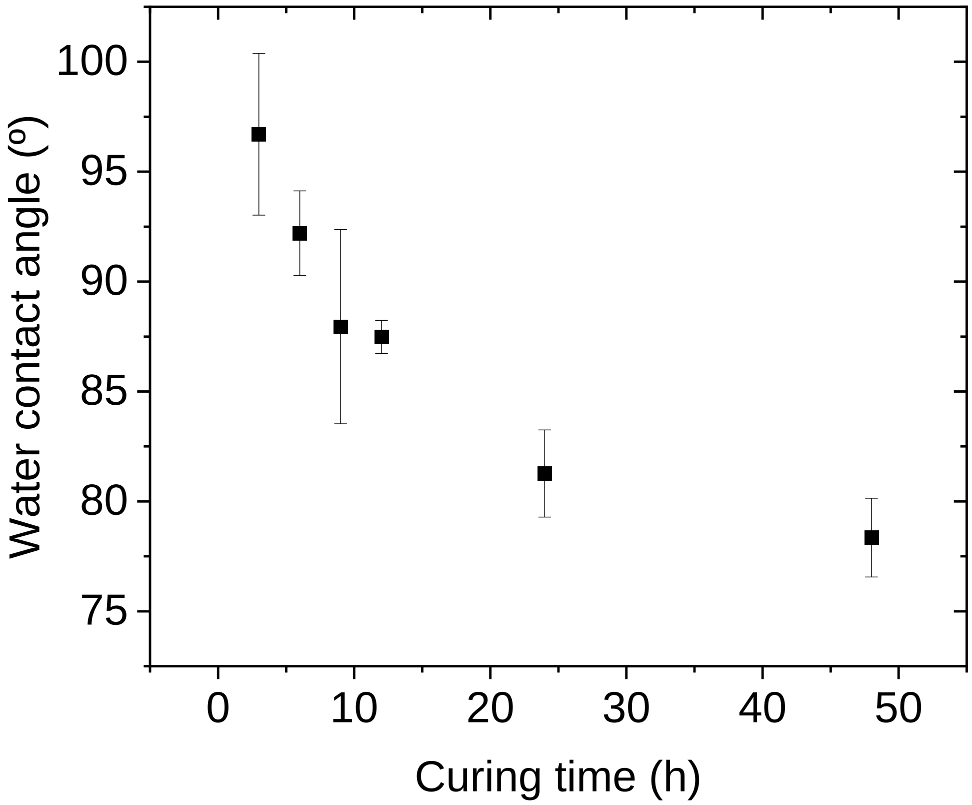

Fig. S9. Water contact angles of suberinic acids thermosets polymerized at 190 ºC for different time lengths, 3 – 48 hours.

Figure S10 shows the weight-based swelling ratio of suberinic acids thermosets in ethanol, THF, water, toluene, and acetone.

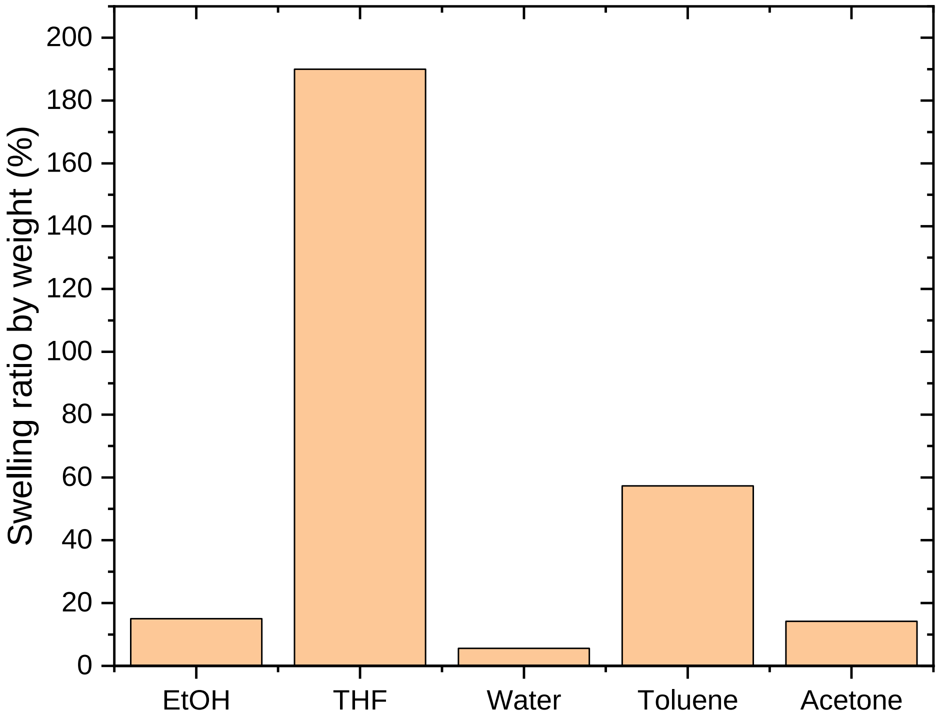

Fig. S10. Swelling ratio (by weight %) of suberinic acids thermosets in ethanol, THF, water, toluene, and acetone.

Figure S11 shows the result of the cross-cut test for examining the paint coatings' resistance to separate from substrates, where no visible coating delamination is found after the test.

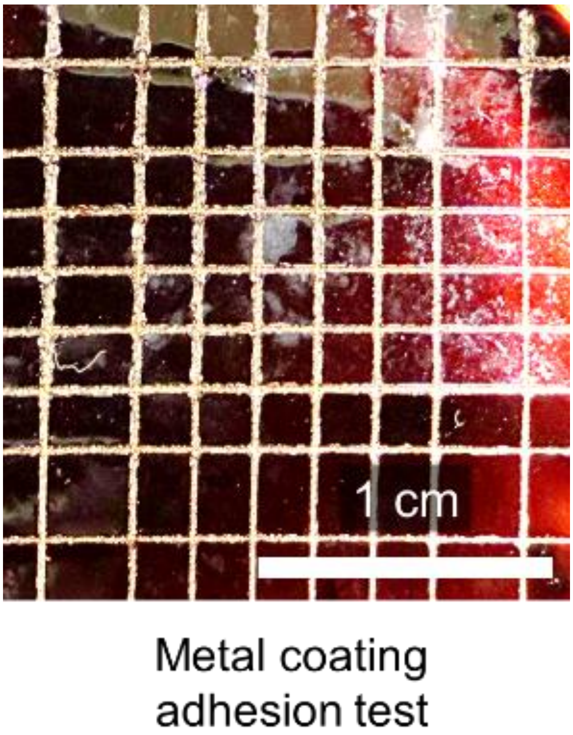

Fig. S11. Digital photograph of cross-cut tested SAs thermoset coated aluminum panel.

Figure S12 shows the stress-strain curves of suberinic acids before recycling (which were polymerized for 4 hours and 5 hours), and after 1st and 2nd times of recycling (3 hours curing and 5 hours curing were tested).

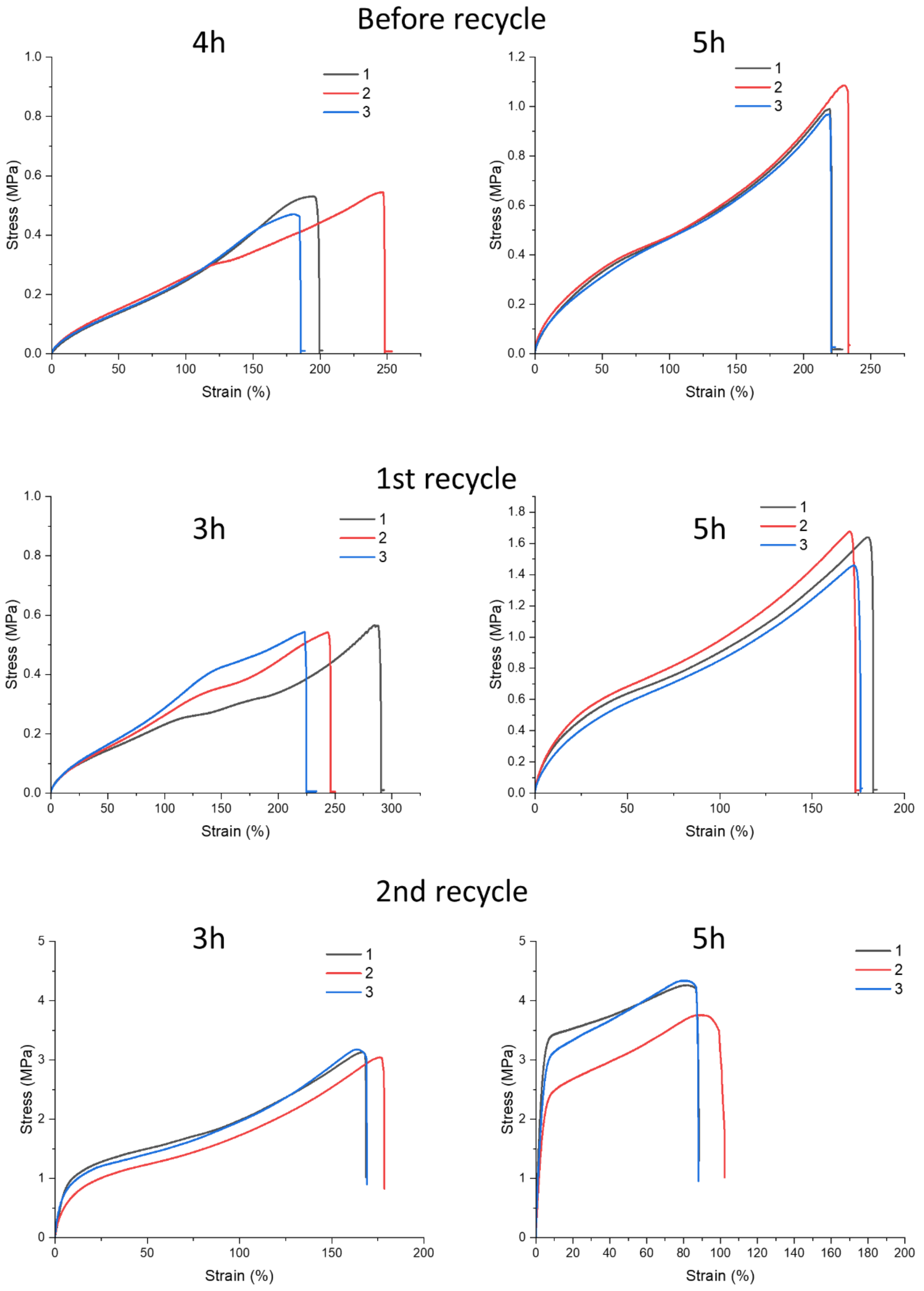

Fig. S12. Stress-strain curves of suberinic acids thermosets before recycle, after 1st recycle and 2nd recycle. Non-recycled samples were cured for 4 or 5 hours, recycled samples were cured for 3 or 5 hours, curing temperature 190 ºC.

Figure S13 shows the stress-strain curves of suberinic acids after 3rd, 4th, and 5th times of recycling (3 hours curing and 5 hours curing were tested).

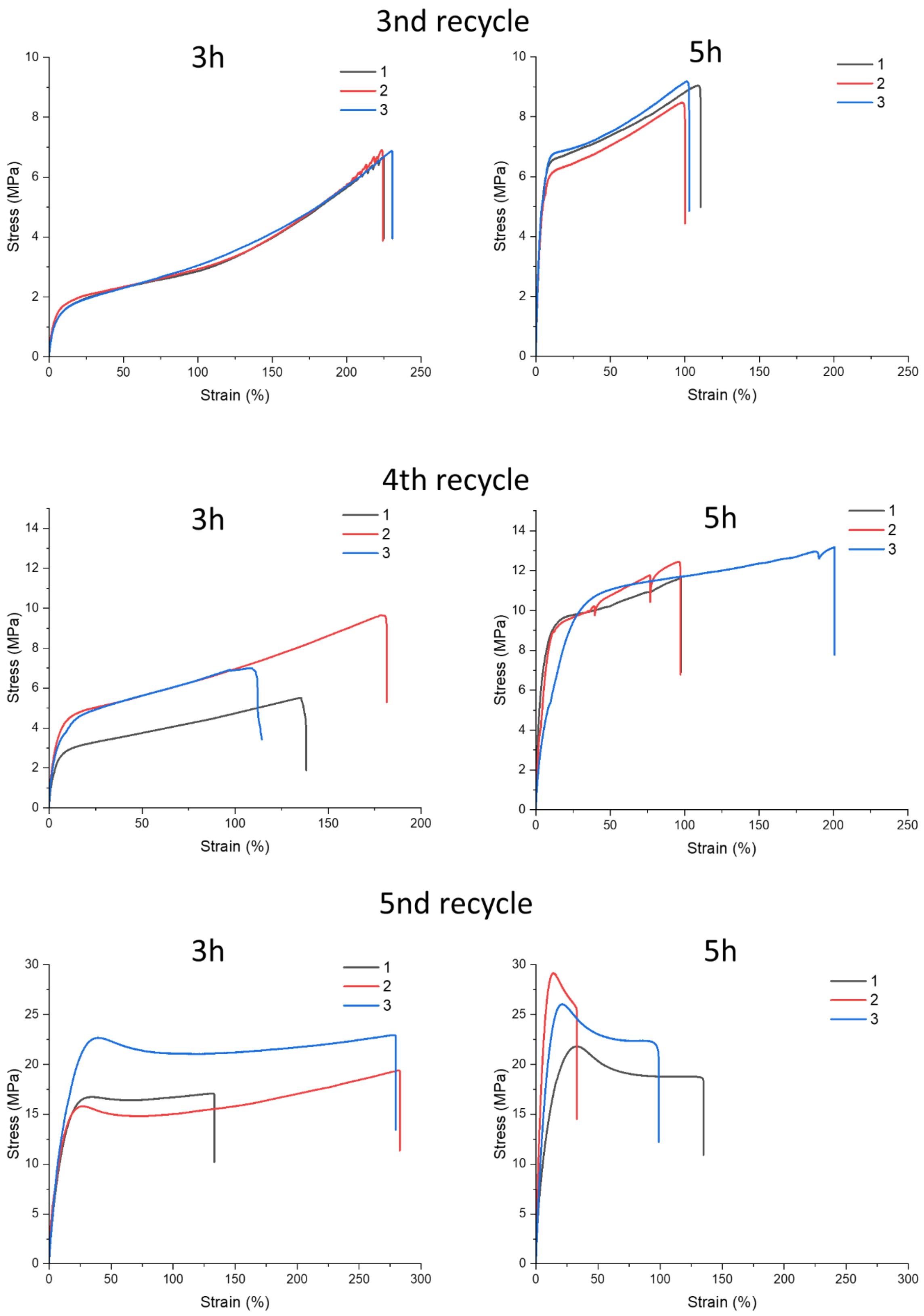

Fig. S13. Stress-strain curves of suberinic acids thermosets after 3rd, 4th, and 5th recycle. Recycled samples were cured for 3 or 5 hours, curing temperature 190 ºC.

Figure S14 shows stress-strain curves of suberinic acids/carbon fiber reinforced polymer (single ply). CFRP is prepared with 20 grams of suberinic acids and 2.5 grams of single-ply carbon fiber fabric.

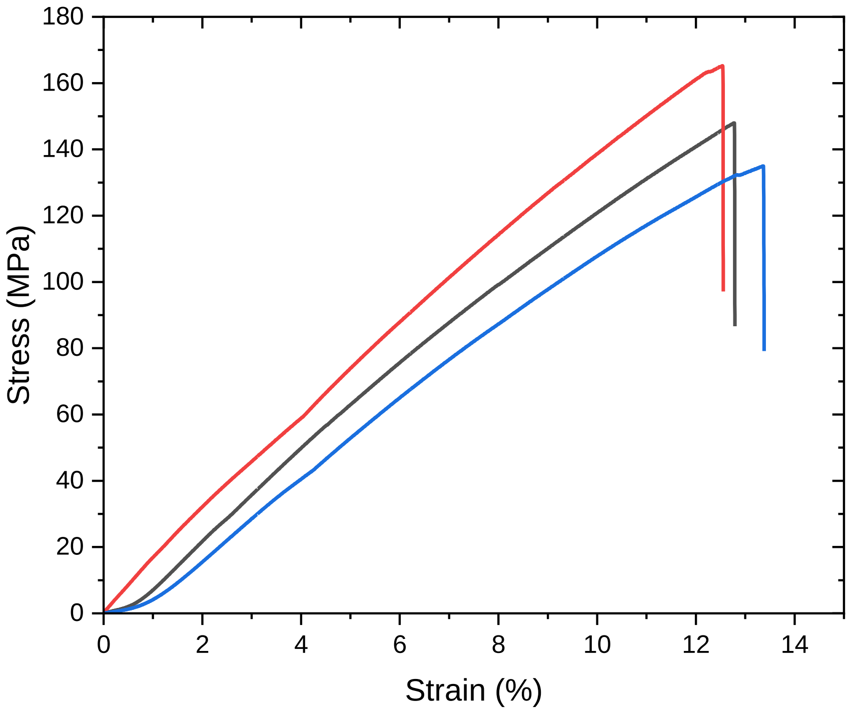

Figure S14. Stress-strain curves of suberinic acids/carbon fiber reinforced polymer.

Figure S15 shows the IR spectra of carbon fiber before and after the recycling process.

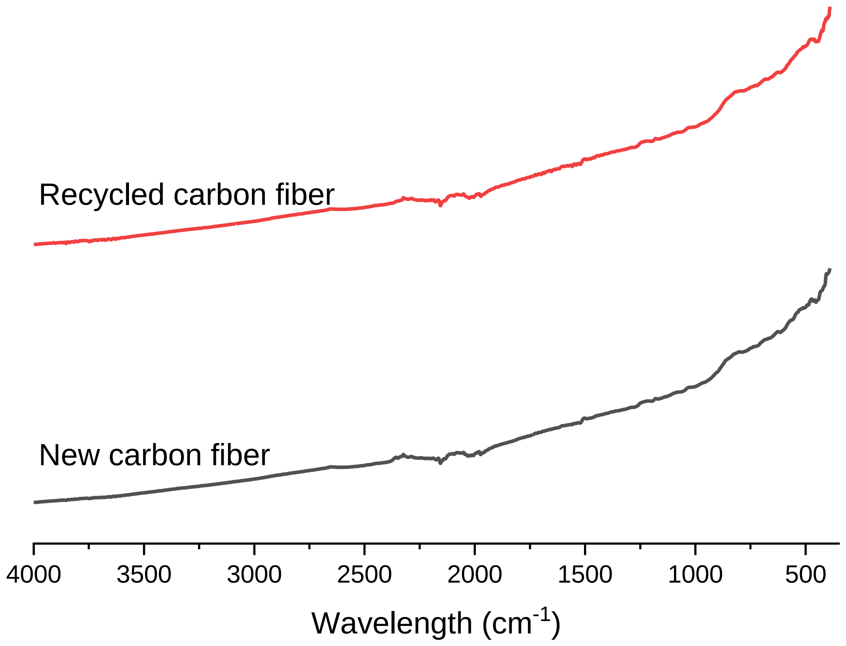

Figure S15. ATR FTIR spectra of the carbon fiber before and after the recycling process.

Figure S16 shows the weight change of suberinic acids/carbon fiber reinforced polymer after 24 hours of immersion in 0.1 M HCl solution, 3 w/v% acetic acid, sunflower oil, and 10% Ethanol. The minimal change in the weight demonstrates great stability of suberinic acids/CFRP.

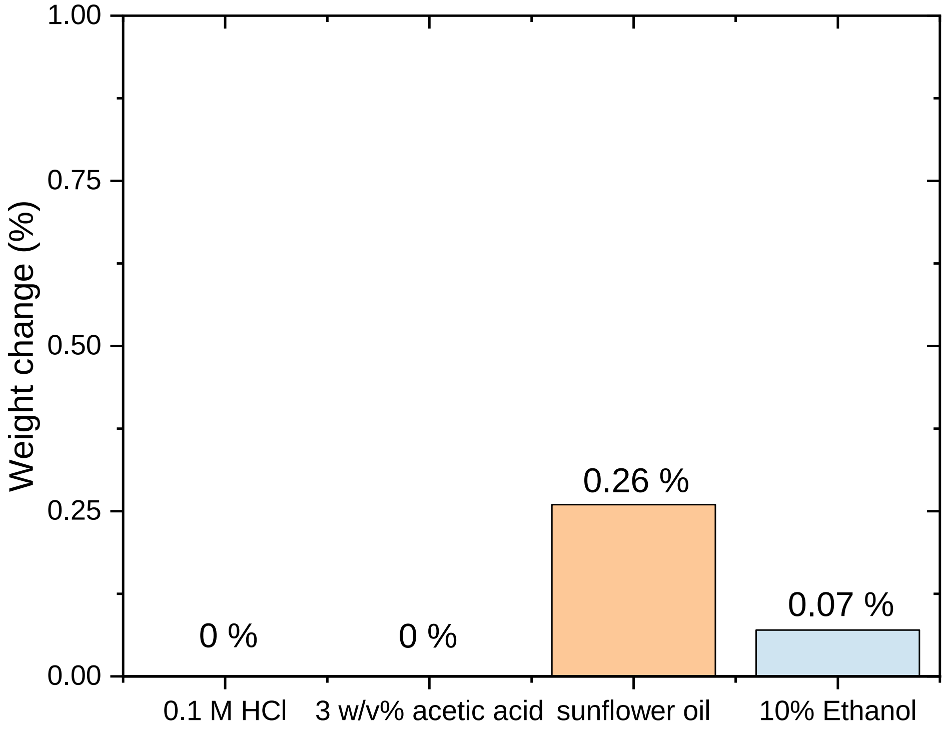

Figure S16. Weight change of suberinic acids/carbon fiber reinforced polymer after 24 hours of immersion in 0.1 M HCl solution, 3 w/v% acetic acid, sunflower oil, and 10% Ethanol.

Table S2 shows the mechanical properties of various polymers used for comparison with the suberinic acids thermosets, where the corresponding figure is shown in Fig. 2. f).

Table S2. Data points used in the property comparison of suberinic acids thermosets with other biobased or synthetic thermosets in **Fig 2. f)**

	Tensile strength (MPa)	Elongation at break
Biobased polyester thermoset[2]	3.2	200%
	31	64%
Suberin-related polyester[3]	1.9	6.7%
Biobased epoxy resin[4]	7.5	28%
	22	18%
Suberin related polyester[5]	1.77	52.2%
Biobased thermoset polyester[6]	62.1	2.4%
	63.3	2.4%
	57	1.6%
Lignin epoxies thermosets[7]	5	44%
Lignin based thermoset[8]	15	10%
	7	90%
	1.3	120%
Lignin+maleic anhydride[9]	40	2.8%
Reprocesssable thermoset[10]	15	8%
Recyclable/reprocessable thermoset[11]	22.3	402%
Recyclable/reprocessable thermoset[11]	32.1	120%
Degradable thermoset[12]	14.5	80%
This work	1.36	373
	9.6	180
	19.4	283
	11.6	96.5
	26	98
	33	22.5

Table S3 shows the corrosion current density and inhibition efficiency of various coating systems as a comparison for the coating demonstrated in the current study.

Table S3. Comparison on the corrosion current density and inhibition efficiency (%) for this study and other biobased or synthetic anticorrosion coating for aluminum.

	Corrosion current density substrate (A.cm2)	Corrosion current density Coated substrate (A.cm2)	Conditions	Inhibition Efficiency (IE%)
Polydopamine based[13]	1.49×10^{-5}	1.8×10^{-8}	Al in 3.5 % NaCl solution	99.9%
Tannic acid based[14]	2.6×10^{-6}	2.2×10^{-7}	AA2024 alloy in 50 mM NaCl solution	91.5%
Lignin based[15]	4.6×10^{-8}	5.05×10^{-10}	Al in 5 % NaCl solution	98.9%
Lignin nanoparticle based[16]	1.35×10^{-4}	2.54×10^{-7}	Al in 5 % NaCl solution	99.8%
Propargylated lignin + epoxy[17]	9.08×10^{-3}	2.13×10^{-5}	Cu in 3.5 % NaCl solution	99.8%
Mxene + PDMS[18]	3.5×10^{-5}	1.49×10^{-9}	Al in 3.5 % NaCl solution	100%
Long chain fatty acids[19]	8.18×10^{-4}	1.48×10^{-6}	Al in 3.5 % NaCl solution	99.8%
Stearic acid based[20]	7.26×10^{-4}	5.01×10^{-5}	Al in 3.5 % NaCl solution	93.1%
Chitosan based[21]	8.56×10^{-6}	8.58×10^{-8}	Cu-Ni (90-10) alloy in 3.5 % NaCl solution	99%
Zn-eggshell particle + starch[22]	2.105×10^{-2}	4.392×10^{-4}	Steel in 3.5 % NaCl solution	97.9%
This study	7.5×10^{-8}	8.3×10^{-13}	Al in 5 % NaCl solution	100%

Table S4 shows the mean -COOH and -OH amount of initial suberinic acids, the hydrolysis product of 48 hours cured SAs thermoset, and the 5th time recycled SAs, which is quantified in mmol g^{-1} by ^{31}P NMR spectroscopy.

Table S4. Mean -COOH and -OH amount in mmol g^{-1} quantified by ^{31}P NMR spectroscopy.

	Mean -COOH amount (mmol g^{-1})	Mean -OH amount (mmol g^{-1})
Initial suberinic acids	2.05	4.24
Hydrolysis product of 48 hours cured SAs thermoset	2.39	3.9
5th time recycled suberinic acids	2.36	5.28

Supplementary references

[1] J. Rizikovs, D. Godina, R. Makars, A. Paze, A. Abolins, A. Fridrihsone, K. Meile, M. Kirpluks, "Suberinic Acids as a Potential Feedstock for Polyol Synthesis: Separation and Characterization" *Polymers 2021, Vol. 13, Page 4380* **2021**, *13*, 4380.

[2] T. J. Lok, J. W. Wong, X. Li, Y. Fu, Y. Xue, F. H. Jamaludin, M. O. Fong, E. B. Edward, C. Ma, S. Chandren, P. S. Goh, T. W. Wong, "Biobased Itaconate Polyester Thermoset with Tunable Mechanical Properties" *Macromolecules* **2024**, *57*, 2317–2328.

[3] H. Garcia, R. Ferreira, C. Martins, A. F. Sousa, C. S. R. Freire, A. J. D. Silvestre, W. Kunz, L. P. N. Rebelo, C. Silva Pereira, "Ex situ reconstitution of the plant biopolyester suberin as a film" *Biomacromolecules* **2014**, *15*, 1806–1813.

[4] C. Menager, N. Guigo, L. Vincent, N. Sbirrazzuoli, "Suberin from Cork as a Tough Cross-Linker in Bioepoxy Resins" *ACS Appl Polym Mater* **2021**, *3*, 6090–6101.

[5] R. Perrotta, I. Kwan, V. Polisetti, M. Ek, A. J. Svagan, M. S. Hedenqvist, "Birch-Bark Suberin-Reconstructed Polyester Film as Packaging Materials" *ACS Sustain Chem Eng* **2025**, DOI 10.1021/ACSSUSCHEMENG.5C02590.

[6] M. A. Hofmann, A. T. Shahid, M. Garrido, M. J. Ferreira, J. R. Correia, J. C. Bordado, "Biobased Thermosetting Polyester Resin for High-Performance Applications" *ACS Sustain Chem Eng* **2022**, *10*, 3442–3454.

[7] C. Gioia, G. Lo Re, M. Lawoko, L. Berglund, "Tunable Thermosetting Epoxies Based on Fractionated and Well-Characterized Lignins" *J Am Chem Soc* **2018**, *140*, 4054–4061.

[8] D. Di Francesco, D. Rigo, K. Reddy Baddigam, A. P. Mathew, N. Hedin, M. Selva, J. S. M. Samec, "A New Family of Renewable Thermosets: Kraft Lignin Poly-adipates" *ChemSusChem* **2022**, *15*, e202200326.

[9] P. Verdross, S. Guinchard, R. T. Woodward, A. Bismarck, "Black liquor-based epoxy resin: Thermosets from untreated kraft lignin" *Chemical Engineering Journal* **2023**, *475*, 145787.

[10] B. Zhang, K. Kowsari, A. Serjouei, M. L. Dunn, Q. Ge, "Reprocessable thermosets for sustainable three-dimensional printing" *Nat Commun* **2018**, *9*, 1–7.

[11] B. Qin, S. Liu, Z. Huang, L. Zeng, J. F. Xu, X. Zhang, "Closed-loop chemical recycling of cross-linked polymeric materials based on reversible amidation chemistry" *Nature Communications 2022 13:1* **2022**, *13*, 1–9.

[12] R. J. Dreiling, K. Huynh, B. P. Fors, "Degradable thermosets via orthogonal polymerizations of a single monomer" *Nature* **2025**, *638*, 120–125.

[13] J. Ou, J. Wang, J. Zhou, S. Liu, Y. Yu, X. Pang, S. Yang, "Construction and study on corrosion protective property of polydopamine-based 3-layer organic coatings on aluminum substrate" *Prog Org Coat* **2010**, *68*, 244–247.

[14] R. del Olmo, A. Bastos, K. Yasakau, A. Sushkova, R. Arrabal, J. Tedim, "Corrosion inhibitor from nature: Fundamentals of tannic acid inhibition for AA2024 alloy" *Appl Surf Sci* **2025**, *680*, 161434.

[15] J. Carlos De Haro, L. Magagnin, S. Turri, G. Griffini, "Lignin-Based Anticorrosion Coatings for the Protection of Aluminum Surfaces" *ACS Sustain Chem Eng* **2019**, *7*, 6213–6222.

[16] A. Moreno, J. Liu, R. Gueret, S. E. Hadi, L. Bergström, A. Slabon, M. H. Sipponen, "Unravelling the Hydration Barrier of Lignin Oleate Nanoparticles for Acid- and Base-Catalyzed Functionalization in Dispersion State" *Angewandte Chemie International Edition* **2021**, *60*, 20897–20905.

[17] I. Pylypchuk, O. Tkachenko, T. Budnyak, M. Sipponen, "Beta-Radiation-Resistant Anticorrosion Coatings Based on Lignin" *Small Science* **2025**, DOI 10.1002/SMSC.202500007.

[18] Y. Zhang, C. Chen, Z. Chen, T. Zhang, Y. Wang, S. Cao, J. Ma, "Superior Anticorrosion Performance of Well-Dispersed MXene-Polymer Composite Coatings Enabled by Covalent Modification and Ambient Electron-Beam Curing" *ACS Appl Mater Interfaces* **2023**, *15*, 11099–11110.

[19] S. K. Pandit, K. Yadav, P. Chauhan, A. Kumar, "Accessing the corrosion resistance for metallic surfaces using long-chain fatty acids" *RSC Applied Interfaces* **2025**, DOI 10.1039/D5LF00125K.

[20] L. Feng, H. Zhang, Z. Wang, Y. Liu, "Superhydrophobic aluminum alloy surface: Fabrication, structure, and corrosion resistance" *Colloids Surf A Physicochem Eng Asp* **2014**, *441*, 319–325.

[21] G. Jena, B. Anandkumar, S. C. Vanithakumari, R. P. George, J. Philip, G. Amarendra, "Graphene oxide-chitosan-silver composite coating on Cu-Ni alloy with enhanced anticorrosive and antibacterial properties suitable for marine applications" *Prog Org Coat* **2020**, *139*, 105444.

[22] V. S. Aigbodion, E. Dinneya-Onuoha, "Unveiling the anti-corrosion properties of Zn-eggshell particle composite coatings on mild steel in seawater-simulated solution using starch as a modifier" *RSC Adv* **2024**, *14*, 24548–24560.
